# Complete Genome Assembly of *Escherichia coli* ATCC 25922, a Serotype O6 Reference Strain

**DOI:** 10.1128/genomeA.00969-14

**Published:** 2014-09-25

**Authors:** T. D. Minogue, H. A. Daligault, K. W. Davenport, K. A. Bishop-Lilly, S. M. Broomall, D. C. Bruce, P. S. Chain, O. Chertkov, S. R. Coyne, T. Freitas, K. G. Frey, H. S. Gibbons, J. Jaissle, C. L. Redden, C. N. Rosenzweig, Y. Xu, S. L. Johnson

**Affiliations:** a Diagnostic Systems Division (DSD), United States Army Medical Research Institute of Infectious Diseases (USAMRIID), Fort Detrick, Maryland, USA; b Los Alamos National Laboratory, Los Alamos, New Mexico, USA; c Naval Medical Research Center (NMRC)-Frederick, Fort Detrick, Maryland, USA; d Henry M. Jackson Foundation, Bethesda, Maryland, USA; e United States Army Edgewood Chemical Biological Center (ECBC), Aberdeen Proving Ground, Maryland, USA

## Abstract

We present the complete genome assembly of *Escherichia coli* ATCC 25922 as submitted to NCBI under accession no. CP009072. This strain was originally isolated from a clinical sample in Seattle, Washington (1946), and is often used in quality control testing. The assembled genome is 5.20 Mb (50.4% G+C content) and includes two plasmids.

## GENOME ANNOUNCEMENT

*Escherichia coli*, a seemingly ubiquitous Gram-negative bacterium, is best known for its ability to cause food-borne outbreaks. The strain ATCC 25922 is a commonly used quality control strain, particularly in antibody sensitivity assays and was originally isolated from a human clinical sample collected in Seattle and WA (1946). It is of serotype O6 and biotype 1. A prior assembly of the genome is publicly available, however, it is in 116 contigs (1).

High-quality genomic DNA was extracted from a purified isolate using a QIAgen Genome Tip-500 at USAMRIID-Diagnostic Systems Divisions (DSD). Specifically, a 100-mL bacterial culture was grown to stationary phase and nucleic acid extracted as per manufacturer’s recommendations. Sequence data generated includes both Illumina (standard unpaired 100-bp library at 300-fold genome coverage) and Roche 454 (7,847- ± 1,962-bp insert, 32-fold genome coverage) technologies (2, 3). Data from the two libraries were assembled together in Newbler (Roche) and the consensus sequences computationally shredded into 2-kbp overlapping fake reads (shreds). The raw reads were also assembled in Velvet and those consensus sequences computationally shredded into 1.5-kbp overlapping shreds (4). Draft data from all platforms were then assembled together with Allpaths and the consensus sequences were computationally shredded into 10-kbp overlapping shreds (5). We then integrated the Newbler consensus shreds, Velvet consensus shreds, Allpaths consensus shreds, and a subset of the long-insert read pairs using parallel Phrap (High Performance Software, LLC). Possible misassemblies were corrected and some gap closure accomplished with manual editing in Consed (6–8).

Automatic annotation for the *E. coli* ATCC 25922 genome utilized an Ergatis based workflow with minor manual curation. The complete annotated genome assembly is available in NCBI (accession no. CP009072) and raw data can be provided upon request. This finished assembly includes one chromosome (5,130,767-bp) and two plasmids (48,488 and 24,185-bp, respectively). Preliminary review of the 5.20-Mbp (50.4% G+C content) genome finds 4,840 coding sequences, 21 rRNAs, and 85 tRNAs.

### Nucleotide sequence accession number.

The annotated genome assembly of *Escherichia coli* ATCC 25922 is available in GenBank under accession no. CP009072.
